# Analysis of T cell repertoires of CD45RO CD4 T cells in cohorts of patients with bullous pemphigoid: A pilot study

**DOI:** 10.3389/fimmu.2022.1006941

**Published:** 2022-11-15

**Authors:** Markus Niebuhr, Farbod Bahreini, Anke Fähnrich, Christina Bomholt, Katja Bieber, Enno Schmidt, Saleh Ibrahim, Christoph M. Hammers, Kathrin Kalies

**Affiliations:** ^1^ Institute of Anatomy, University of Lübeck, Lübeck, Germany; ^2^ Lübeck Institute of Experimental Dermatology, University of Lübeck, Lübeck, Germany; ^3^ Department of Dermatology, University of Lübeck, Lübeck, Germany

**Keywords:** naive and memory CD4 T cells, autoimmunity, skin diseases, T cell receptor, repertoire diversity, next generation sequencing

## Abstract

Autoimmune diseases develop over years - starting from a subclinical phenotype to clinically manifest autoimmune disease. The factors that drive this transition are ill-defined. To predict the turning point towards clinical disease and to intervene in the progress of autoimmune-mediated dysfunction, the establishment of new biomarkers is needed. Especially CD4 T cells are crucially involved in autoimmunity: first, during the initiation phase, because they lose their tolerance towards self-peptides, and second, by the subsequent ongoing presentation of self-peptides during the active autoimmune disease. Accordingly, changes in the degree of diversity of T cell receptor (TCR) repertoires in autoimmunity have been reported. These findings led to the hypothesis that transition from pre-disease to autoimmune disease is associated with an increase of abnormally expanded T cell clones that occupy large portions of the TCR repertoire. In this pilot study, we asked whether the ratio and the diversity of the TCR repertoires of circulating memory (CD45RO) and naïve (CD45RA) CD4 T cells could serve as a predictive factor for the development of autoimmunity. To find out, we analyzed the TCRβ repertoires of memory and naïve CD4 T cells in a small cohort of four gender- and age-matched elderly patients having the autoimmune blistering disease bullous pemphigoid or non-melanoma skin cancers. We found that the extent of clonal expansions in the TCRβ repertoires from the circulating memory and naïve CD4 populations did not differ between the patient groups. This result shows that the diversity of TCR repertoires from peripheral CD4 T cells does not reflect the manifestation of the skin-associated autoimmune disease BP and does not qualify as a prognostic factor. We propose that longitudinal TCR repertoire analysis of younger patients might be more informative.

## Introduction

The T cell receptor (TCR) repertoire is the sum of all T-cell antigen-binding sequences that are present within one individual. It is determined not only by the number of different TCR combinations but also by the number of T cells within one body. Recent data predicted 4 x 10^11^ circulating T cells in humans (1) with a potential diversity of 10^6^ -10^8^ different αβ TCR combinations (2, 3). A highly diverse TCR repertoire is the prerequisite for (i) mediating protection against the broad variety of pathogenic antigens, (ii) eliminating altered self to control neoplasias, and (iii) preventing recognition of self-antigens to cause autoimmunity. The diversity of the TCR repertoire changes during a lifetime. It is at its peak during young ages due to the constant thymic output of recently developed naïve T cells, and it decreases during aging as a result of the thymic involution, increased homeostatic proliferation, and the development of a T cell memory pool (4–6). Along with this shrinking diversity, the tendency to acquire inflammatory autoimmune diseases rises with aging. This holds true also for autoantibody-mediated diseases due to the prominent role of CD4 T cells in providing B cell help (7, 8). For example, the titers of antinuclear antibodies and rheumatoid factors increase after the age of 60 years in systemic lupus erythematosus (SLE) and rheumatoid arthritis (RA), respectively. There is growing evidence that the clinical manifestations of autoimmune diseases correlate with changes in features of the TCR repertoire (9). Thus, it has been shown that not only the diversity of the TCR repertoire is significantly decreased in SLE patients but also the number of disease-associated T cell clones connects with disease severity in SLE and RA patients (10, 11). Additionally, the onset of clinical features in cases of SLE and RA correlates to changes in the specificity of the autoantibodies (12–14). The reasons for the shift in autoantibody-specificity and for turning the preclinical stage into clinical autoimmunity are not known. Considering the relationship between the TCR repertoire, aging, and the development of autoimmune diseases, we hypothesize that the diversity of the TCR repertoire might change profoundly in the process of development of the autoimmune disease from preclinical autoimmunity and could serve as predictive factor.

To find out we compared the peripheral TCRβ repertoire of small cohorts of four autoimmune patients and four patients with a non-autoimmune disease. Specifically, we focused on the circulating memory CD4 T cell population, and hypothesized that the degree of TCRβ repertoire diversity of this population might be lower in autoimmune patients when compared to non-autoimmune patients. Memory CD4 T cells emerge upon repeated contacts with their antigenic peptide presented in the MCHII complex. They are defined as lymphocytes that present fast recall responses to antigens and thereby provide immediate protection in peripheral tissues in case of pathogenic invasions. Thus, memory T cells are irreplaceable in combating infections and preventing pathogen-specific reinfections. However, if T cells lose their tolerance to self-peptides and differentiate into memory T cells, they bear the dangerous potential to induce autoimmunity. In contrast to pathogens, self-peptides cannot be dispelled, which leads to ongoing contacts between T cells and self-peptides resulting in chronic inflammations and might lead, as hypothesized in this study, to an accumulation of memory T cell clones specific to self-peptides. This contrasts with naïve T cells that exhibit no immediate effector functions because of the lack of antigen contacts and extensive clonal divisions (15). Memory T cells can be distinguished from naïve T cells by the expression of the surface markers CD45RO (CD45RAneg). CD45RO and CD45RA (CD45RApos) are isoforms of the CD45 tyrosine phosphatase, which is expressed on all T cells. Upon activation, T cells change the surface expression from the CD45RA to the CD45RO isoform. Therefore, CD45RO T cells are considered as memory T cell population including both recently activated effector and memory T cells (16, 17).

As autoimmune patient group, we chose patients diagnosed with the autoimmune blistering disease bullous pemphigoid (BP), which is mediated by autoantibodies directed against domains of type XVII collagen in the skin (18, 19). As control group, age- and gender-matched patients with non-melanoma skin cancers (NMSC) such as basal (BCC) and squamous skin cancer (SCC) were selected. By choosing NMSC patients as control group we focused on the availability of samples from elderly patients, who (i) were diagnosed with a completely different disease entity (autoimmune versus cancer), (ii) displayed inflammatory skin conditions, and (iii) were hospitalized at the same ward, to exclude potential effects of the surrounding microbiome. NMSC is characterized by a low metastatic rate from the host cells of the skin without involvement of autoreactive and systemic T and B cell responses (20, 21) (**Supplementary Table 1**). This contrasts with the inflammatory skin disease BP, which is induced by aberrant T and B cell responses including an accumulation of CD4 and CD8 T cells in skin lesions (22–25). In addition, skin lesions in BP patients appear body-wide, which indicates the systemic character of this autoimmune disease (18, 19).

In this study, we compared the number of T cells within the memory and naïve CD4 T cells population between both patient groups. Deep sequencing was used to identify their TCRβ repertoires for enumeration of TCRβ clonotypes and to compare the degree of the TCRβ repertoire diversity, the clonal overlap between the memory and naïve CD4 populations, and other TCR repertoire features such as the V/J gene usage and the complementarity determining region 3 (CDR3) length of the antigen-binding regions. Unexpectedly, despite differences in the ratio of CD4 T cell numbers and TCRβ clonotypes, we found that the extent of clonal expansion of the circulating memory CD4 T cell population did not differ between the patient groups.

## Material and methods

### Patient samples

Patients with BP and age- and sex-matched biopsy-confirmed patients with NMSC were recruited at the University of Luebeck. The peripheral venous blood from participants was collected in the EDTA-containing tubes and the skin biopsy was taken as a punch biopsy and shock frozen in liquid nitrogen. The blood from 14 BP patients and six NMSC patients was used to enumerate naïve and memory CD4 T cells. Finally, sorted naïve and memory CD4 T cells from 4 BP and 4 NMSC patients as age- and gender-matched controls were subjected to deep sequencing for analysis of their TCRβ repertoire. BP was diagnosed based on the typical clinical manifestation of the disease, detection of the linear deposition of IgG at the dermal-epidermal junction in the direct immunofluorescence, detection of IgG on the blister roof on salt-split skin biopsies, or the enzyme-linked immunosorbent assay against BP180-NC16A (data not shown). The clinical characteristics of the BP and NMSC patients are summarized in **Supplementary Table 1**. The study was conducted based on the principles of the 1964 Helsinki declaration and its further amendments. All patients enrolled in this study provided written informed consent prior to their inclusion in this study. The study was approved by the ethical committee of the University of Luebeck (Approval ID: AZ 12-178).

### Isolation of CD45RA positive and CD45RA negative CD4 T cells

Peripheral blood mononuclear cells (PBMC) were extracted from 9 ml EDTA blood samples using a Ficoll density gradient separation (Ficoll-Paque Plus; GE Healthcare Bio-Science, Sweden). The CD4 T cells were then negatively selected from the PBMC *via* a magnetic separation using a CD4 T cell isolation kit (Miltenyi Biotec, Germany) according to the manufacturer’s protocol. The flow-through (CD4 T cells) were magnetically labeled with CD45RA MicroBeads. CD45RO (CD45RAneg) memory CD4 T cells were isolated as flow-through by negative selection and CD45RA CD4 T cells were released from microbeads based on the manufacturer’s instruction (Miltenyl Biotec, Germany). An aliquot of the isolated cells was then stained using DAPI (4′,6-Diamidin-2-phenylindol; Thermo Fisher Scientific, USA), FITC-conjugated anti-human CD45RA (Clone HI100, Biolegend, USA), PE-conjugated anti-human CLA (Clone HECA-452, Miltenyl Biotec, Germany), PerCP/Cy5-conjugated anti-human CD4 (Clone RPA-T4, Biolegend, USA), APC-conjugated anti-human CD45RO (Clone UCHL1, Biolegend, USA) following standard procedures, and fluorescence-activated cell sorting measurements were performed on the MACSQuant X (Miltenyi Biotec, Germany) Flow Cytometer. Sample analysis was performed using FlowJo v10.8.1 (BD Biosciences). CD45RO and CD45RA CD4 T cells were identified as CD4/CD45RO co-expressing cells and CD4/CD45RA co-expressing cells, respectively.

### Identification of TCRβ clonotypes

Total RNA isolation was performed for CD45RA CD4, CD45RO CD4 T cell populations, and the skin biopsy sample with the innuPREP RNA Mini Kit (Analytik Jena, Hildesheim, Germany). The cDNA preparation and the amplification of the antigen-binding site (CDR3β region) of the TCRβ chain were conducted as suggested by the manufacturer (iRepertoire, patent 7999092, 2011, Huntsville, USA) and prepared for pair-end sequencing using an Illumina Miseq system as previously reported (26). The MiXCR pipeline (v4.0) was used for the processing, alignment, assembling, correcting PCR errors, and exporting the data from the fastq files (27, 28). TCRβ clonotypes were then annotated as described before (29, 30). The TCRβ repertoire was further analyzed by employing the immunarch package (version 0.6.9) on the R programming language (R-4.4.1) (31). The diversity of TCRβ repertoire was estimated using the Inverse Simpson Index. The Jaccard Index was used to quantify the overlapping TCRβ clonotypes by normalizing their numbers. Both indices were calculated with the immunarch package. To avoid unpredictable PCR and sequencing errors, the default parameters (“eliminate these errors”) were used. Additionally, to avoid artificial diversity due to PCR errors, all TCRβ clonotype sequences that appeared only once were removed. The number of total and unique TCRβ clonotypes are shown in **Supplementary Table 2**.

### Statistical analysis

Statistical analyses were carried out using either R programming language or GraphPad Prism (Version 9.0, GraphPad Software Inc, USA). Statistical significance was evaluated by 2-way ANOVA with turkeys’ multiple comparison test or Mann-Whitney U tests. The data were considered statistically significant at p values < 0.05.

## Results

### Quantification of circulating memory and naïve CD4 T cell populations

In this study, we hypothesize that the clonal abundances of memory CD4 T cells would differ between autoimmune (BP) and non-autoimmune patients (NMSC). Peripheral blood (9ml) was collected for analyzing the peripheral TCRβ repertoire of circulating T cells. Because BP is a disease of the elderly, the average age of both groups is about 87 ± 2.5 years. Most patients show several comorbidities and are given long-term medications (**Supplementary Table 1**).

**Figure 1:**
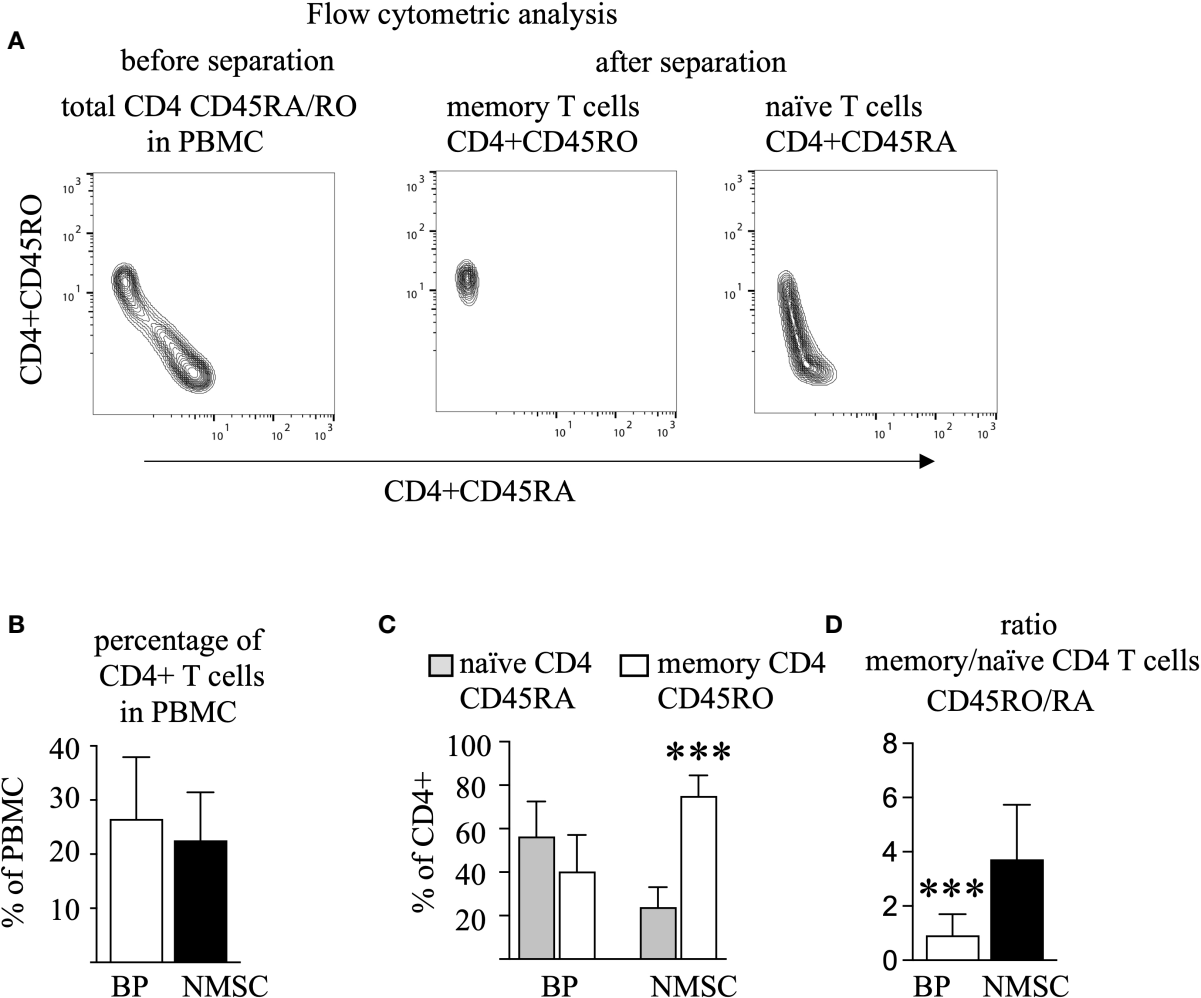
The number of memory T cells prevails in NMSC patients. PBMC from BP and NMSC patients were sorted for CD4 CD45RA and CD45RO T cells. **(A)** The gating strategy of one typical example is shown. Because CD45RO T cells were sorted by negative selection (middle panel), they appear as a specific population in contrast to the CD45RA population (right panel). **(B)** The size of the circulating CD4 T cell population is similar in BP and NMSC patients as shown as percentages of PBMC (Data are mean ± SD, p=0.43, not significant (n. s.) Mann-Whitney test). **(C)** NMSC patients harbor significantly more CD45RO T cells (Data are mean ± SD, ***p < 0.001, 2-way ANOVA with turkeys’ multiple comparison test). **(D)** The ratio of the CD45RO/RA population is significantly lower in BP patients. (Data are mean ± SD, ***p < 0.001, Mann-Whitney U test), (in **B–D**, n=14 (BP) and n=6 (NMSC)).

To identify memory CD4 T cells, CD4 and CD45RO T cells were sorted by two negative selection steps, after which the CD45RO CD4 T cells are clearly identifiable as distinct cell populations by flow cytometric analysis (**Figure 1A**, middle panel). All other CD4 T cells, which did bound to the magnetic beads that were coated with CD45RA-directed antibodies were defined as the CD45RA population. For the sake of simplicity and easier reading these CD45RA CD4 T cells are called naïve T cell population, even though this population is a heterogeneous mixture that contains CD4 T cells that are highly positive or almost dull for CD45RA (**Figure 1A**, right panel) (32). In all samples, the sum of the CD4 CD45RO and CD4 CD45RA was close to 100% (data not shown). Evaluation of cell numbers and percentages revealed that approximately 30% of the PBMC were CD4 positive and did not differ between the BP group and the NMSC group (**Figure 1B**). This was different for the CD45RA/RO populations. Here, we found that BP patients have almost equal numbers of memory and naïve CD4 T cells in contrast to NMSC patients, in which a significantly higher proportion of memory T cells was found. Calculation of the ratio of CD45RO to CD45RA shows a significant difference between BP patients and NMSC patients (**Figures 1C, D**).

### No difference in the abundances of TCRβ clonotypes between the patient groups

**Figure 2:**
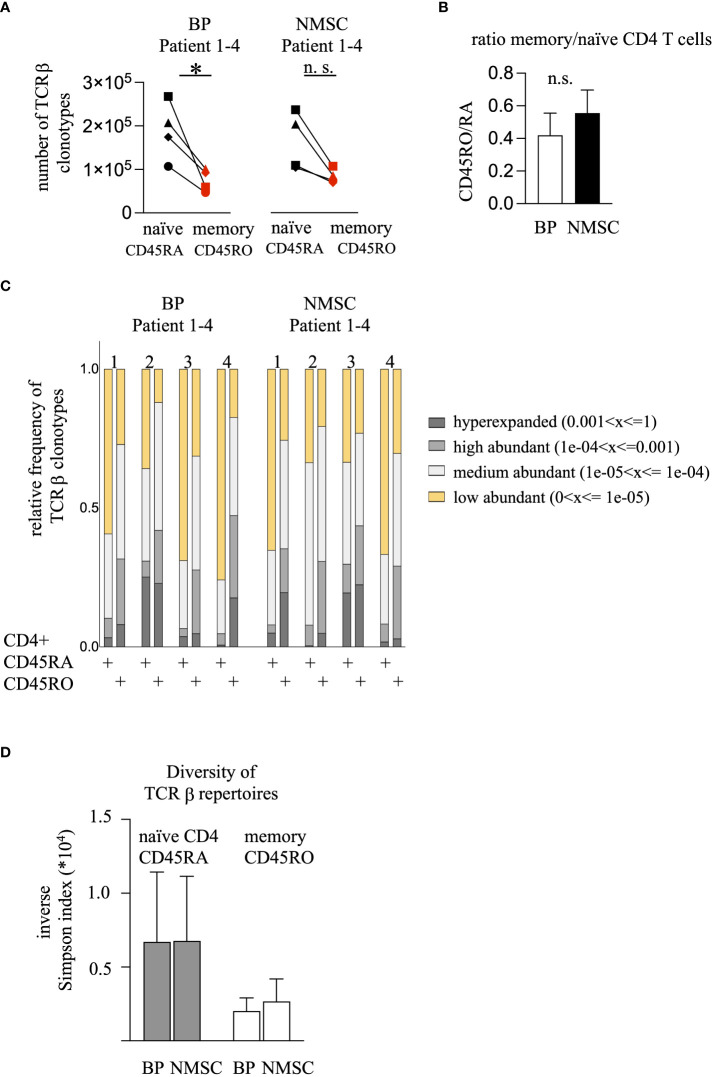
BP patients harbor fewer CD45RO clonotypes but the frequency of the high-abundant TCRβ clonotypes do not differ compared to the NMSC patients. **(A)** The numbers of the unique TCRβ clonotypes from the CD45RA (black symbols) and CD45RO (red symbols) CD4 T cell population are shown. The values obtained from the same patient are connected. (n = 4, *p<0.05, One-way ANOVA with turkeys’ multiple comparison test). **(B)** The ratio of the unique TCRβ clonotypes from the CD45RO/RA population was calculated. Data are mean ± SEM. No significant difference between BP and NMSC patients was found. (n = 4, Mann-Whitney test). **(C)** The clonal distribution of the TCRβ clonotypes is shown. TCRβ clonotypes were divided regarding their clonal abundance. Each bar represents the clonotypes of the CD45RA and CD45RO population from one patient. Each bar is divided into 4 sections. Each section represents the fraction of clonotypes that exist at a certain frequency (yellow: > 1 x 10^-5^, bright gray: 1 x 10^-5^ - 1 x 10^-4^, gray: 1 x 10^-4^ - 1 x 10^-3^, dark gray: < 1 x 10^-3^). **(D)** Inverse Simpson Index shows no difference between the BP or NMSC patients. (Data are mean ± SEM, gray bars: CD45RA, white bars: CD45RO no significance, 2-way ANOVA).

For initial repertoire analysis, memory and naïve CD4 T cell populations from four typical patients of each group were chosen for identification of their CDR3 antigen-binding sequences. RNA was extracted from each T cell population, their TCRβ genes were amplified by multiplex PCR and the nucleotide sequence was identified by next-generation sequencing using the MiSeq™ Illumina system. Between 46’714 to 267’496 (mean ± SD = 127696 ± 67540) unique TCRβ clonotypes could be identified (**Supplementary Table 2**). Thereby, the memory CD4 T cell sample harbored less TCRβ clonotypes compared to the naïve CD4 T cell population in each patient regardless of the type of pathogenesis (**Figures 2A, B**). This difference in clonotype numbers might be caused by the enormous number of naïve CD4 T cells that never expanded due to the lack of antigen and therefore exist at low frequencies. Indeed, apportioning the TCRβ repertoires according to their clonal abundance in four groups (hyperexpanded > 0.001, high abundant 0.0001-0.001, medium abundant 0.00001-0.0001, and low abundant < 0.00001) revealed that the naïve population contained the highest number of low abundant clonotypes (yellow bars, Fig. 2C). Vice versa, the memory population is characterized by a higher number of hyperexpanded and highly abundant clonotypes (gray bars, **Figure 2C**). To quantify these observations, the Inverse Simpson Index was calculated that measures both the number of different TCRs and their clonal expansion. Due to the different numbers of TCRβ clonotypes per patient the values are variable. However, a trend towards a decreased Inverse Simpson Index in the memory populations could be observed, which indicates the higher number of expanded TCRβ clonotypes in this population compared to the naïve populations (**Figure 2C**). No difference was found between the patient groups (**Figure 2D**). This data contrasts with our hypothesis that the CD4 memory population of autoimmune patients would display more abundant clonotypes.

### Similarity analysis and individual clone tracking reveal a high overlap between memory and naïve CD4 TCRβ clonotypes

**Figure 3:**
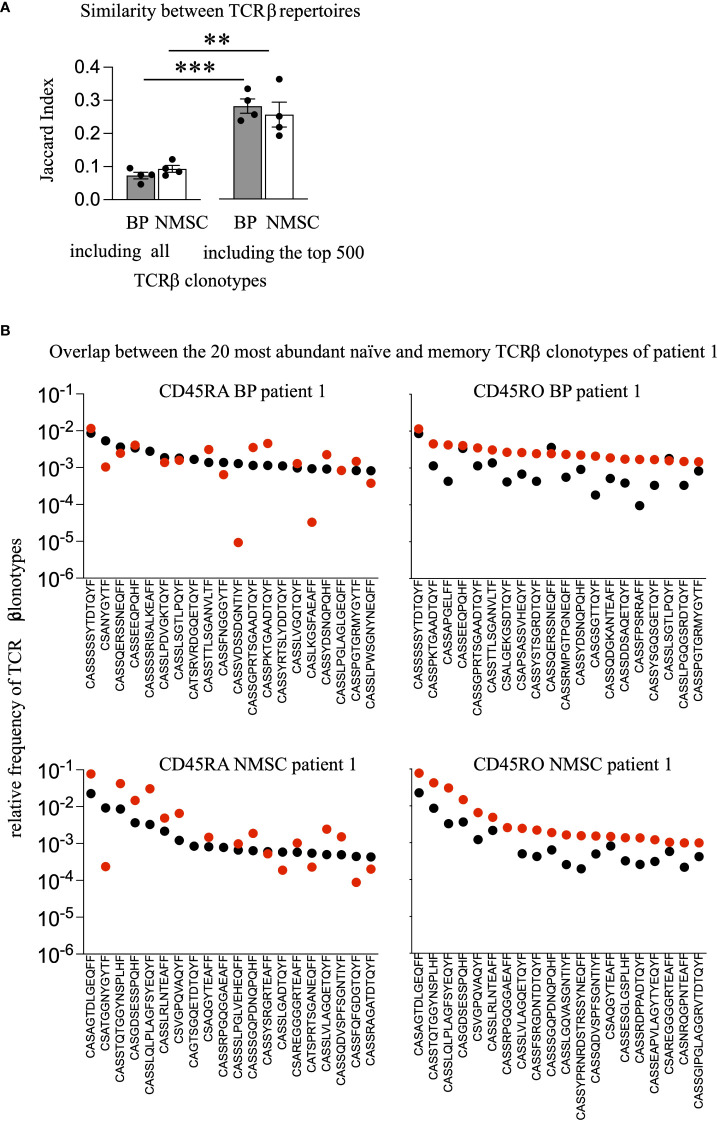
No difference in the degree of overlapping TCRβ clonotypes between BP and NMSC patients. **(A)** To assess the portion of overlapping TCRβ clonotypes between the CD45RO and CD45RA populations within each patient quantitatively, the Jaccard Index was calculated by either including all TCRβ clonotypes or including only the top 500 most abundant TCRβ clonotypes. (Data are mean ± SEM, ***p < 0.001, **p < 0.01, 2-way ANOVA with turkeys’ multiple comparison test). **(B)** The 20 most frequent TCRβ clonotypes present in the CD45RO population (black dots, left panel) are compared to their presence in the CD45RA population (red dots, left panel) and vice versa, the 20 most frequent TCRβ clonotypes present in the CD45RA population (black dots, right panel) are compared to their presence in the CD45RO population (red dots, right panel) within BP patient 1 (upper panel) or NMSC patient 1 (lower panel). One representative comparison out of all patients is shown.

**Figure 4:**
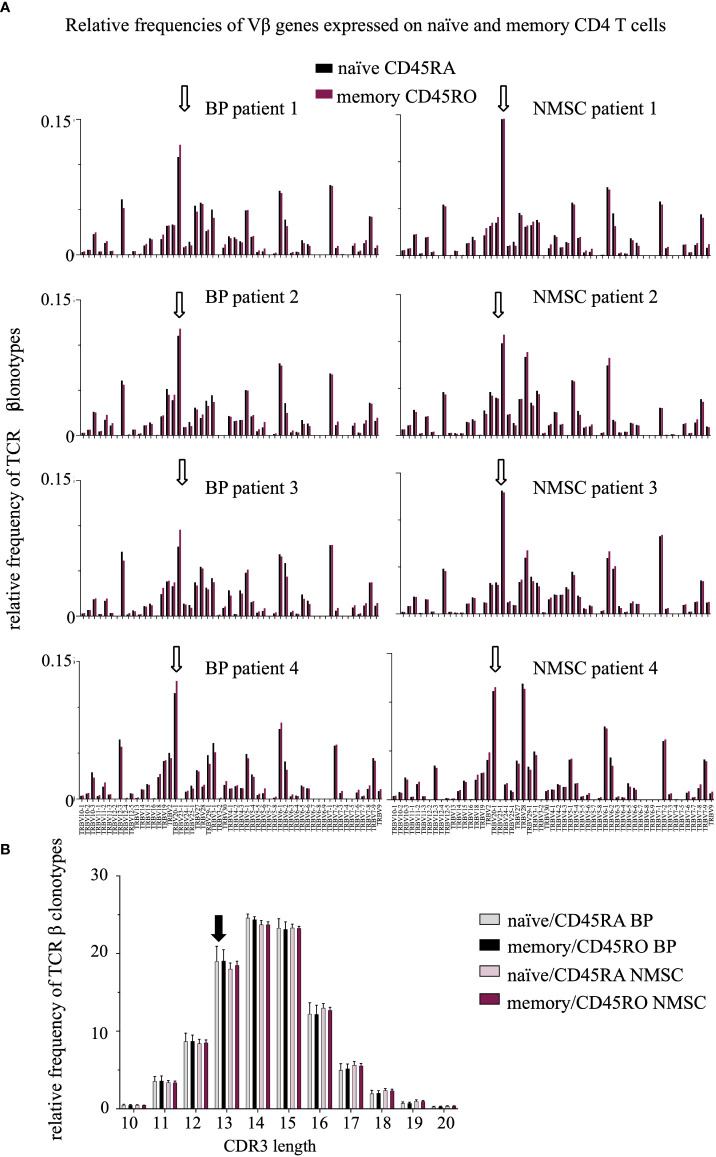
A trend toward a reduced length of the CDR3 region in BP patients was observed but no difference in the V gene usage between the patient groups. **(A)** Relative frequencies of Vβ genes were compared between the CD45RO (red bars) and CD45RA (black bars) population in BP patients (1-4, left) and NMSC patients (1-4, right). White arrows indicate the high expression of the Vβ genes TRB20-1 in all samples. The high usage of TRBV20-1 has been described previously and was associated with aging (35, 36). **(B)** Relative length distribution of the CDR3 regions of CD45RO and CD45RA TCRβ clonotypes were compared between BP and NMSC patients (Data are means ± SD). The black arrow indicates the increased frequency of TCRβ clonotypes with a CDR3 length of 13 amino acids in BP patients (black, gray bars) compared to NMSC patients (bright and dark red bars).

Potential explanations for the lack of more abundant TCRβ clonotypes in the autoimmune group could be the presence of effector memory expressing CD45RA (terminally differentiated helper T lymphocytes, Temra) cells, which form by the conversion of CD45RO into CD45RA T cells (33, 34). To address a potential conversion of CD45RO to CD45RA T cells we identified the overlapping T cell clonotypes in both populations, within each patient. As shown in **Figure 3A**, the Jaccard Index, which reflects the similarity between samples based on the number of overlapping clonotypes after normalization to the overall number of clonotypes, revealed no significant differences between BP and NMSC patients. The identified means are 0.09 and 0.07 for the BP and NMSC patient group, respectively, when compared within one patient (intra-individually, **Figure 3A**). Naturally, since each patient has its individual TCR repertoire, these values are lower when compared to all other patients of the same group (inter-individually) (**Figure 4A**). By limiting the comparison of overlapping TCRβ clonotypes to the top 500 most expanded TCRβ clonotypes from the memory and naïve populations within one patient, the Jaccard Index increased significantly regardless of the patient group (**Figure 3A**). Strikingly, the top 20 most abundant TCRβ clonotypes from memory and naïve CD4 T cell population overlap almost completely within each patient. **Figure 3B** shows the presence and abundance of the top 20 TCRβ clonotypes from the CD45RA population within the CD45RO population of one typical patient (**Figure 3B**, left panel) and vice versa (**Figure 3B**, right panel). The data obtained for all other patients were comparable (data not shown). In conclusion, the high number of overlapping TCRβ clonotypes raises the possibility that memory CD4 T cells are converted to Temra cells by re-expressing CD45RA. However, there is no difference between the patient groups. Considering that this is just a pilot study with four individuals per group, further analysis with more detailed phenotypically selected CD4 cell subsets will be required to understand the high similarity between both T cell populations.

### Characteristics of TCRβ repertoire: TRBV, TRBJ gene usage, and CDR3 length

**Figure 5:**
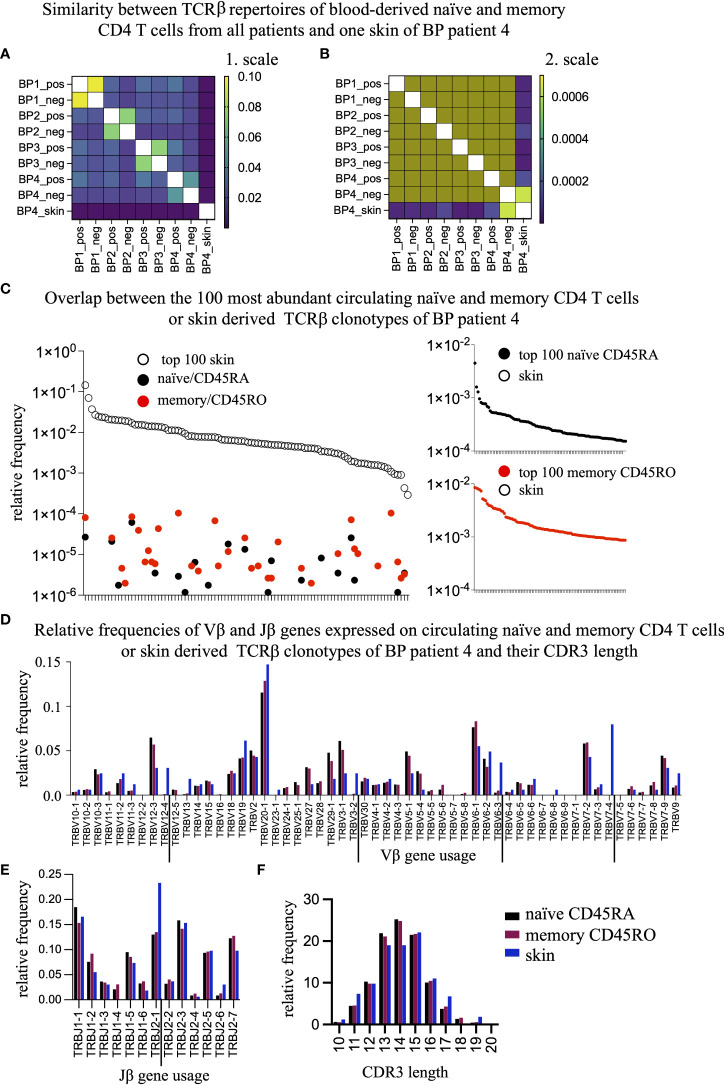
The overlap between the skin-residing and the circulating TCRβ clonotypes is negligible. **(A, B)** To assess the percentage of overlapping TCRβ clonotypes between the blood-derived CD45ROand CD45RA populations and the skin-resident T cell clones of BP patient 4, the Jaccard Index was calculated and displayed as a heat map with a range from 1e-006 to 0.1 **(A)** or from 1e-006 to 0.0007 **(B)**. **(C)** The top 100 most frequent TCRβ clonotypes present in the skin of BP4 (white dots, left panel) are compared to their presence in the CD45RA (black dots, left panel) or CD45RO population (red dots, left panel) of BP patient 4. Vice versa, the top 100 most frequent TCRβ clonotypes of in the CD45RA population (black dots, right upper panel) or the CD45RO population (red dots, right lower panel) are compared to their presence in the skin and could not be found. **(D, E)** Relative frequencies of Vβ genes **(D)** and Jβ genes **(E)** were compared between the skin (blue bar), the CD45RO (red bars), and CD45RA (black bars) populations of BP patient 4. The TRBV genes and TRVJ genes that are preferentially expressed in the skin are underlined. Indicated are means ± SEM. **(F)** Relative length distribution of the CDR3 regions of skin-residing TCRβ clonotypes were compared to the CD45RO and CD45RA populations of BP patients 4. (Data are means ± SEM). Differences can be observed at a length of 11, 13, 14, and 17 amino acids.

This high overlap between the intra-individual memory and naïve CD4 T populations suggests that typical TCRβ repertoire features such as V/J usage and CDR3 length would be also highly similar within each patient. Indeed, most of the V genes were expressed in similar frequencies in both CD4 T cell populations within each patient and regardless of the type of disease (**Figure 5A**). Unexpectedly, also the pattern of the V/J gene expression was similar in all patients. For example, TCR beta variable gene (TRBV) 20-1 was expressed at a high frequency in all samples. Comparable results were found for the J genes (**Supplementary Figure 1**).

Studying the CDR3 length distribution revealed no difference between both CD4 T cell populations (**Figure 4B**). Instead, subtle shifts were observed between BP and NMSC patients. The TCRβ clonotypes of BP patients are in tendency one amino acid shorter than those of the NMSC controls.

In conclusion, our pilot study shows that neither the extent of clonal expansion nor the overlap of TCRβ clonotypes from circulating memory and naïve CD4 T cells differ between the groups of autoimmune and non-autoimmune patients. Obviously, in contrast to our hypothesis, the memory CD4 T cell population was not expanded under autoimmune conditions.

### TCRβ clonotypes in skin lesions overlap only marginally with the circulating CD4 T cell populations of the same donor

Next, we asked whether the circulating TCRβ clonotypes would be present in the skin lesions of BP patients (**Figure 1C**, **Figure 2A**). Because only one of the BP patients (BP4) agreed to provide a skin biopsy simultaneously with the collection of blood our analysis was restricted to this one sample. In the perilesional biopsy of BP4, we found 163 unique TCRβ clonotypes (**Supplementary Table 2**). As shown in **Figure 5A**, the Jaccard Index-based heat map reveals that the skin biopsy of BP4 has very low similarity to the circulating memory and naïve CD4 T cell populations from all patients. For better visualization, we changed the color codes (**Figure 5B**). Now, it is obvious that the TCRβ clonotypes from the skin and the memory CD4 T cells overlap highest in the same donor BP4 when compared to the T cell populations of all other patients (bright green square in the lower right corner, **Figure 5B**). Taking a closer look at the top 100 most abundant skin-derived TCRβ clonotypes, it becomes evident that they are present at very low frequencies only in the circulation irrespective of the memory or naïve population (between 0.01% and 0.0001% of the blood TCRβ repertoire, **Figure 5C**, left panel). Even more so, the top 100 most abundant circulating TCRβ clonotypes do not exist in the skin lesions at all (**Figure 5C**, CD45RA-derived clonotypes right, upper panel; CD45RO-derived clonotypes, right lower panel), which could suggest a skin-specific accumulation. In line, the TRBV and TRBJ gene usage from TCRβ clonotypes of the skin lesion and of circulating CD4 T cell population are different (**Figures 5D, E**). Especially, T cells expressing the TRBV genes 12-4, 3-2, 6-3, and 7-4 and the TRBJ gene 2-1, reside in the skin lesion but are only minor in blood. Moreover, the length of the CDR3 regions differs between blood- and skin-derived TCRβ clonotypes from the same patient (**Figure 5F**). These data indicate that T cell clones distribute differently between skin lesions and blood.

## Discussion

This is the first study evaluating and comparing the circulating CD45RO (memory) and CD45RA (naïve) CD4 T cells in BP patients at the level of TCRβ clonotypes. We hypothesized that a persistent T cell activation under autoimmune condition would lead to a higher portion of high-abundant T cell clones in the circulating memory CD4 T cell population of BP patients when compared to age- and gender-matched non-autoimmune (NMSC) controls. Our data show that this is not the case (**Figure 2**). Furthermore, other features of TCR repertoires such as V/J gene usage have not been affected in BP patients (**Figure 4**). This contrasts with other autoimmune diseases such as SLE, RA, and type 1 diabetes mellitus, for which a reduced diversity of the TCR repertoire from the circulating CD4 T cells pool and changes in the V/J gene usages have been reported ( (11) reviewed in (9)). These discrepancies might be caused by technical differences, but also by natural reasons such as the stage of the disease activity, the types of medication, and the type of the autoimmune disease. Another important difference between the above-mentioned studies of TCR repertoires in SLE, RA, and type 1 diabetes mellitus is the age of the patients. The average age of the BP patients in this study is 87 ± 2.5. Profound changes in the T cell repertoire have been described in individuals, who are older than 70 years (4). It is assumable that at older ages, the extent of the clonal expansions of the most abundant T cell clones might have such a high level, that any distinction between autoimmune and non-autoimmune patients is not assessable anymore. Further analysis will be required not only by including more patients but also by including additional markers such as CD62L, CCR7, CD27, and CD28 for detailed phenotyping. In line, comparative analysis of the TCR repertoire should be extended to include CD8 T cells, gamma-delta T cells, and especially follicular T helper cells in BP and NMSC patients. For example, a crucial role for CD8 T cells has been reported for pemphigus vulgaris (37). Gamma-delta T cells are involved in the pathogenesis of BP and pemphigus vulgaris (38, 39).

Besides the main finding that the extent of clonal expansion was not elevated in the BP group compared to the NMSC group (**Figures 2B, C**), we found that the ratio between circulating memory and naïve CD4 T cells was significantly lower in the BP group compared to the NMSC patients. The means of the ratios are 0.9 and 3.7 for BP and NMSC, respectively (**Figures 1B, C**). It is tempting to speculate that these simple ratios in cell numbers could serve as a prognostic factor during the development of autoimmunity. Longitudinal studies should answer this question in further analysis. Opposite results have been found in other autoimmune diseases, such as RA and SLE, and also in healthy donors (32, 40, 41). The question arises what causes the decreased ratio of memory to naïve CD4 T cells in BP patients? BP-specific CD45RO CD4 T cells have been isolated from the peripheral T cell pool of patients, which does not exclude that the majority of this population might reside in the skin (42–44). In line with the decreased ratio of memory to naïve CD4 T cells in BP patients, the ratio of the TCRβ clonotypes is also lower in BP patients. This difference was not so obvious anymore (**Figure 2A**), which might be due to the normalization of the RNA concentrations for all samples that were capped at 0.5 µg according to the protocol for library preparation in both T cell populations (iRepertoire, Inc., Huntsville, USA).

The identification of overlapping TCRβ clonotypes between the memory and naïve population revealed an extensive overlap within each patient (**Figure 3**) and a highly similar pattern of the V/J gene usages (**Figure 4**). Especially the top 500 TCRβ clonotypes overlap between both CD4 T cell populations. Considering the well-established antigen-specificity of the memory CD4 T cell population, these data are difficult to interpret. The process of CD4 T cell activation is a multistep process (45), during which CD4 T cells lose their expression of CD45RA upon contact with antigen (16, 17). Thus, one would expect that TCR sequences from memory and naïve CD4 T cells would differ due to the proposed antigen-specificity of the memory T cell population. It will be interesting to find out whether this overlap reflects the presence of Temra cells, which re-express CD45RA and represent a terminal stage of effector differentiation (46). Especially, when considering the old age of both patient groups one could speculate that this high overlap between memory and naïve TCRβ clonotypes might be caused by conversions to Temra cells and might be typical for repertoires of the elderly. In line, a recent longitudinal study reported that the number of overlapping CD4 TCRβ and TCRα clonotypes increases with age (6). Additionally, the presence of high abundant TCR clonotypes in naïve T cell populations and their maintenance by homeostatic proliferation might contribute (8, 47). It is particularly noteworthy that especially the naïve CD4 population is not pure and contains transient double positive cells CD45RA+CD45RO+ (32) even though MACS sorting was performed carefully according to the manufacturer’s protocol (Miltenyi Biotec). More selective markers such as CD27 or CD28 should be applied in further studies.

Finally, we tested whether T cells would preferentially accumulate in skin lesions of BP patients. Comparison of TCRβ sequences of the one skin that matched donor and time point of blood collection revealed a low similarity to the blood sample (**Figure 5B**). This finding agrees to the differential accumulation of T cell bearing TRBV 12-4, 3-2, 6-3, and 7-4 and the TRBJ 2-1 segment between skin and blood, which might not be surprising (**Figures 5D–F**). Considering that the collected blood samples reflect only 1-2% of the circulating blood volume and that up to 50% of memory CD4 T cells reside in lymphoid organs and do not enter the circulation (44), it is unlikely to find overlapping clonotypes. However, it is important to note that in this patient BP4, the similarity between the skin and memory T cell repertoires is higher than between skin and naïve T cell repertoires even though the naïve population harbors substantially more TCRβ clonotypes (**Figure 5B**). This opens the possibility of a skin-specific TCR repertoire in BP patients. A recent analysis of TCR repertoires revealed skin-specific T cell accumulations in healthy human skins. In line, skin-specific TCR repertoires have been described in the mouse model for pemphigoid diseases (22, 43). On the other side, it has been shown that topical treatments affect the number of BP reactive T cells in the blood, which suggests ongoing exchanges of T cells between blood and skin (24). Further approaches are required to assess the role of skin-immigrating T cells even though it might be difficult to recruit enough patients. Understandably, the willingness of the patients to donate biopsies from inflamed perilesional skin is rather low.

In summary, aberrant T cell responses have been reported in BP, even though it is an autoantibody-mediated disease. This pilot study shows that the ratio of circulating memory CD4 T cells to naïve CD4 T cells is lower in BP patients than in controls. Comparison of the TCRβ repertoires of these circulating T cell populations revealed no differences in diversity, the extent of clonal expansion, and V/J gene usage between the BP and NMSC patients. This data indicates that an enumeration of memory and naïve T cell subsets in BP patients might serve as prognostic parameters and should be followed up, especially in longitudinal studies.

## Data availability statement

The datasets presented in this study can be found in online repositories. The names of the repository/repositories and accession number(s) can be found below: https://www.ncbi.nlm.nih.gov/, bioproject # PRJNA867683.

## Ethics statement

The studies involving human participants were reviewed and approved by The ethical committee of the University of Luebeck. The patients/participants provided their written informed consent to participate in this study.

## Author contributions

CH, ES, KK, MN, SI contributed to conception and design of the study. CB, FB, MN, KB performed experiments. AF, KK, MN, FB analyzed the data. CH, ES recruited patients. KK, SI obtained funding. FB, KK wrote the manuscript. All authors contributed to the article and approved the submitted version.

## Funding

This study was funded by the German Research Foundation (DFG) within the framework of the Schleswig-Holstein Excellence Cluster I&I (EXC 306, Inflammation at Interfaces, project XTP4), the graduate schools GRK 1727/2 and GRK 2633/1, the TR-SFB654 project C4 at the University of Lübeck. We acknowledge financial support by the Land Schleswig-Holstein within the funding program Open Access Publikationsfonds.

## Acknowledgments

We thank L. Gutjahr, P. Lau, R. Pagel and D. Rieck for their technical assistance.

## Conflict of interest

The authors declare that the research was conducted in the absence of any commercial or financial relationships that could be construed as a potential conflict of interest.

## Publisher’s note

All claims expressed in this article are solely those of the authors and do not necessarily represent those of their affiliated organizations, or those of the publisher, the editors and the reviewers. Any product that may be evaluated in this article, or claim that may be made by its manufacturer, is not guaranteed or endorsed by the publisher.

## Abbreviations

BCC, basal cell carcinoma; BP, bullous pemphigoid; CD3, complementarity determining region 3; CD45RO CD45RA negative, memory; CD45RA, CD45positive, naïve; NMSC, non-melanoma skin cancers; PBMC, peripheral blood mononuclear cells; RA, rheumatoid arthritis; SCC, squamous cell carcinoma; SLE, systemic lupus erythematosus; TCR, T cell receptor; TRBJ, TCR beta joining genes; TRBV, TCR beta variable genes.
