# Supplementary material for: Analysis of T cell repertoires of CD45RO CD4 T cells in cohorts of patients with bullous pemphigoid: A pilot study
Source: Front Immunol. 2022 Nov 15;13:1006941. doi: 10.3389/fimmu.2022.1006941 (PMC9706093; doi:10.3389/fimmu.2022.1006941)
Supplement: Supplementary file 1 [file Table_1.docx]

**Supplementary Table 1: Clinical characteristics of patients**

| **Patient/Control** | **Sex** | **Born (year)** | **Skin condition** | **Other diseases** | **Sample** | **Medication** | **Diagnosis** |
| --- | --- | --- | --- | --- | --- | --- | --- |
| BP1 | M | 1928 | BP | Diabetes, renal insufficiency, hypertension, cerebral infarction | CD45RO, CD45RA | Class IV topical glucocorticoids, Azathioprine, Cetirizine | ELISA for BP180 NC16A |
| BP2 | M | 1934 | BP | Diabetes, cerebral infarction, heart disease | CD45RO, CD45RA | Class II topical glucocorticoids, class IV topical glucocorticoids | ELISA for BP180 NC16A |
| BP3 | M | 1934 | BP | Cerebral infarction, myocardial infarction | CD45RO, CD45RA | Dapsone, class IV topical glucocorticoids, Desloratadine | ELISA for BP180 NC16A |
| BP4 | F | 1932 | BP | MGS, hypothyreosis, coronary heart disease | CD45RO, CD45RA Skin | Dapsone, class IV topical glucocorticoids | ELISA for BP180 NC16A |
|  |  |  |  |  |  |  |  |
| NMSC1 | M | 1929 | BCC, actinic keratoses | Chronic heart disease | CD45RO, CD45RA |  |  |
| NMSC2 | M | 1935 | Actinic keratoses, SCC | Chronic heart disease | CD45RO, CD45RA |  |  |
| NMSC3 | M | 1933 | BCC, actinic keratose, SCC | Chronic heart disease,  melanoma (?) | CD45RO, CD45RA |  |  |
| NMSC4 | F | 1934 | SCC, BCC, M. Bowen | Microscopic polyangiitis (pANCA pos.), Diabetes mellitus | CD45RO, CD45RA |  |  |

Abbreviations: MGS, BCC, basal cell carcinoma; SCC squamous cell carcinoma; pANCA, BP180, NC16A
