# Supplementary material for: Analysis of T cell repertoires of CD45RO CD4 T cells in cohorts of patients with bullous pemphigoid: A pilot study
Source: Front Immunol. 2022 Nov 15;13:1006941. doi: 10.3389/fimmu.2022.1006941 (PMC9706093; doi:10.3389/fimmu.2022.1006941)
Supplement: Supplementary file 2 [file Table_2.docx]

**Supplementary Table 2: Total and unique CDR3β sequences obtained from**

**9 ml EDTA Blood and one skin biopsy**

| CD45RO | total TCRβ clonotypes (x10^6^) | unique TCRβ clonotypes |
| --- | --- | --- |
| BP1 | 1.298265 | 92274 |
| BP2 | 1.388796 | 46714 |
| BP3 | 1.540575 | 101611 |
| BP4 | 1.526158 | 59677 |
| NMSC1 | 1.296206 | 85583 |
| NMSC2 | 1.327736 | 68887 |
| NMSC3 | 1.326042 | 74100 |
| NMSC4 | 1.715456 | 106776 |

| CD45RA | total TCRβ clonotypes (x10^6^) | unique TCRβ clonotypes |
| --- | --- | --- |
| BP1 | 1.031226 | 174467 |
| BP2 | 1.319964 | 107096 |
| BP3 | 1.313883 | 207420 |
| BP4 | 1.713748 | 267496 |
| NMSC1 | 1.173569 | 202867 |
| NMSC2 | 1.448991 | 108898 |
| NMSC3 | 1.70121 | 103572 |
| NMSC4 | 1.562657 | 235705 |

| perilesional skin | total TCRβ clonotypes (x10^6^) | unique TCRβ clonotypes |
| --- | --- | --- |
| BP4 | 1.270798 | 163 |

**Supplementary Table 2.** CDR3β identification, clonotype clustering, and correction of PCR and sequencing errors were performed using MiXTCR software according to the IMGT nomenclature (27). To avoid unpredictable PCR and sequencing errors, the default parameters (“eliminate these errors”) were used.
