# Supplementary figures and images for: Analysis of T cell repertoires of CD45RO CD4 T cells in cohorts of patients with bullous pemphigoid: A pilot study
Source: Front Immunol. 2022 Nov 15;13:1006941. doi: 10.3389/fimmu.2022.1006941 (PMC9706093; doi:10.3389/fimmu.2022.1006941)

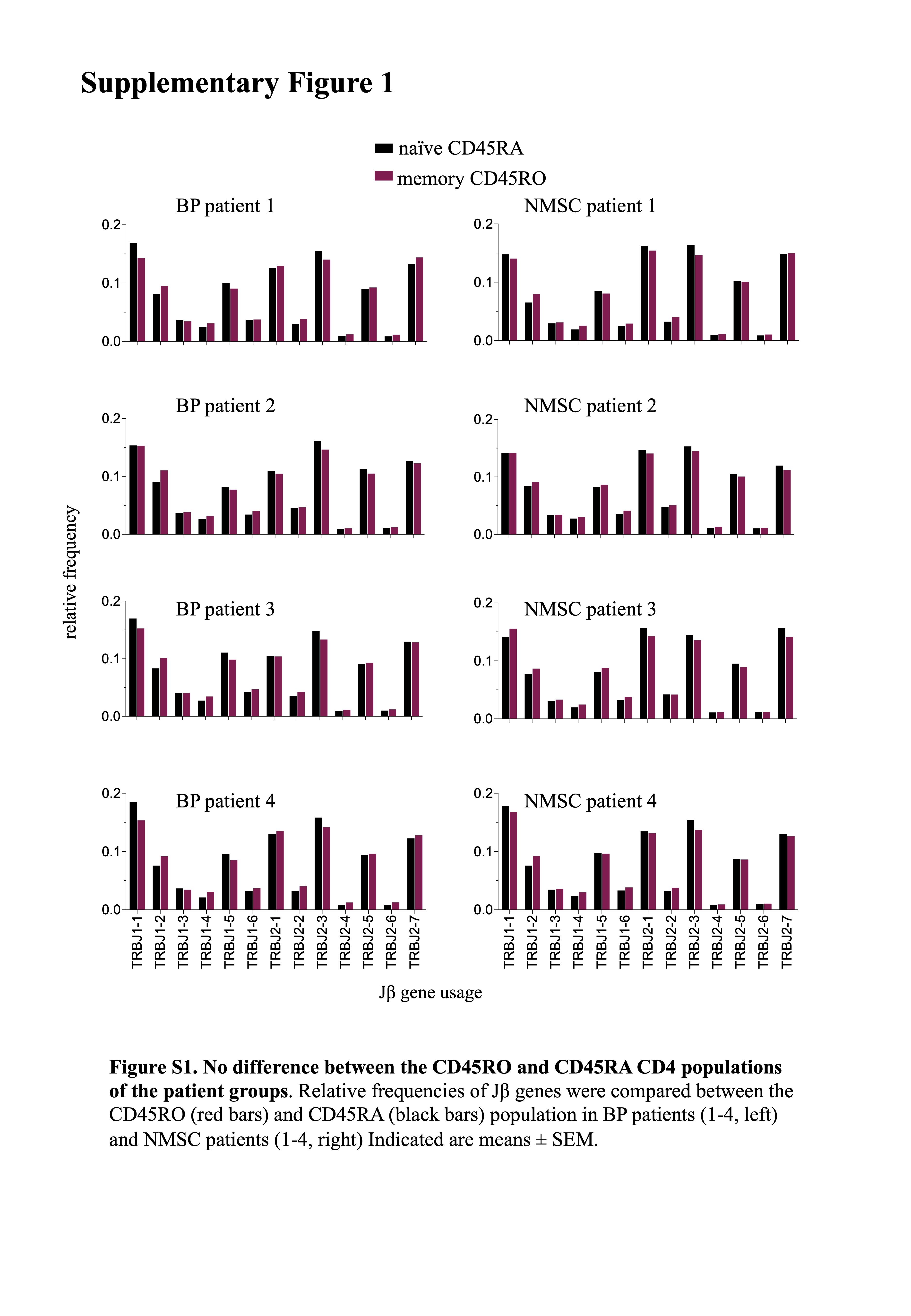

Supplement: Supplementary file 3 [file Image_1.jpg]
